# Infected Mature Teratoma in the Anterior Mediastinum Removed Using the Da Vinci Robotic System

**DOI:** 10.7759/cureus.27919

**Published:** 2022-08-12

**Authors:** Manar Edriss, Eve Paxton, Kevin Jamil

**Affiliations:** 1 Surgery, Wayne State University School of Medicine, Detroit, USA; 2 General Surgery, Beaumont Hospital, Dearborn, USA; 3 Thoracic Surgery, Oakland University William Beaumont School of Medicine, Dearborn, USA

**Keywords:** diaphragm injury, mediastinum, minimally invasive, robotics, teratoma

## Abstract

Mature teratomas have been found to be the most common type of extragonadal primary germ cell tumors found in the anterior mediastinum. Over the past decade, several reports have been published using minimally invasive approaches to remove mediastinal masses. Of these publications, only one reported a teratoma excision from the anterior mediastinum via the Da Vinci Robot. Additionally, there have been few reports regarding teratomas infected with bacteria. This is a case of a 37-year-old man with an incidentally identified *Proteus mirabilis* infected mature teratoma in the anterior mediastinum that was removed with the Da Vinci Robotic System.

## Introduction

A teratoma is a germ cell tumor composed of various types of somatic tissue originating from different germinal layers including the ectoderm, mesoderm, and endoderm. Due to their embryologic origin, it is common for teratomas to contain a variety of somatic tissue types including hair, teeth, cartilage, etc. Subtypes of teratomas include mature and immature teratomas. Unlike immature teratomas, mature teratomas have a rare malignant potential occurring in 1% of all cases [1]. Mature mediastinal teratomas account for 60% of all mediastinal germ cell tumors [2]. Traditional treatment consists of a complete excision via median sternotomy, although a thoracosternotomy may be required for giant tumors [2]. Due to increased morbidity, operations have evolved to video-assisted thoracoscopic surgery (VATS) and now robotic-assisted thoracoscopic surgery (RATS). Literature focusing on RATS for the removal of teratomas in the anterior mediastinum is lacking [3]. In addition, it is highly unusual that teratomas located in the mediastinum are found to be infected. A review of the literature reveals only one other case of an infected teratoma in this location, which was positive for Salmonella [4].

## Case presentation

**Figure 1:**
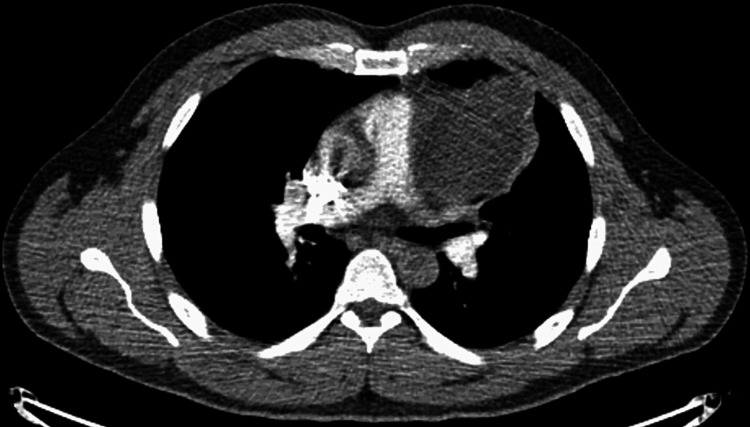
Anterior mediastinal mature teratoma

A 37-year-old man presented to the ED with neck and abdominal pain secondary to a motorized bike accident that occurred two days prior. The patient underwent a CT scan of the chest, abdomen, and pelvis with IV contrast, which was negative for any acute traumatic processes but incidentally found a large 7.9 cm mass in the anterior mediastinum and thoracic surgery was consulted (Figure 1). At the time the patient denied dyspnea, stridor, chest palpitations, fevers, or weight loss. The patient received a two-day course of empiric antibiotics prior to a CT-guided biopsy of the anterior mediastinal mass, which demonstrated purulent appearing aspirate fluid. The cyst was aspirated to completion. The cultures grew Proteus mirabilis and pathology demonstrated tissue consistent with a mature teratoma. Da Vinci-assisted left-sided thoracic mediastinal mass resection was scheduled.

**Figure 2:**
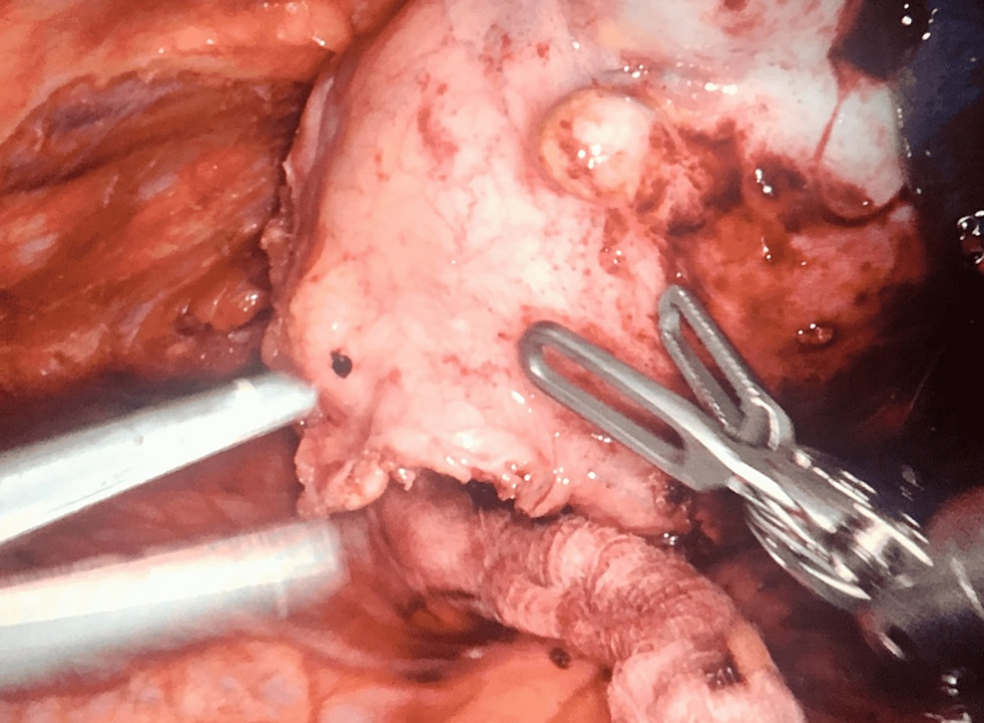
Intraoperative photo of mature teratoma

In the operating room after dual lumen ET tube intubation, right lateral decubitus positioning, and superficial skin preparation, the trocars were placed and the Da Vinci XI platform was docked using three arms and instruments were placed in standard direct vision. The mass was visualized and the adhesions to the left upper lobe were partially teased before a decision was made to resect a small portion of the lung due to the strong lung adhesions to the mass. A robotic blue load Endo-GIA XI Da Vinci stapler was used to resect the mass. Thymic fat was then incised along the pericardium at the inferior aspect of the mass. An intimate association of the mass to the phrenic nerve was observed with an inflammatory reaction complicating resection of the mass. To avoid cautery in close proximity to the phrenic nerve, bipolar cautery and careful dissection along the nerve were used. The nerve was placed on a significant stretch due to the large size of the mass. The mass was successfully detached from the phrenic nerve and no transection of the nerve occurred throughout the operation (Figure 2). A large tributary to the innominate vein was found and dissection was carried out using a vessel sealer.

**Figure 3:**
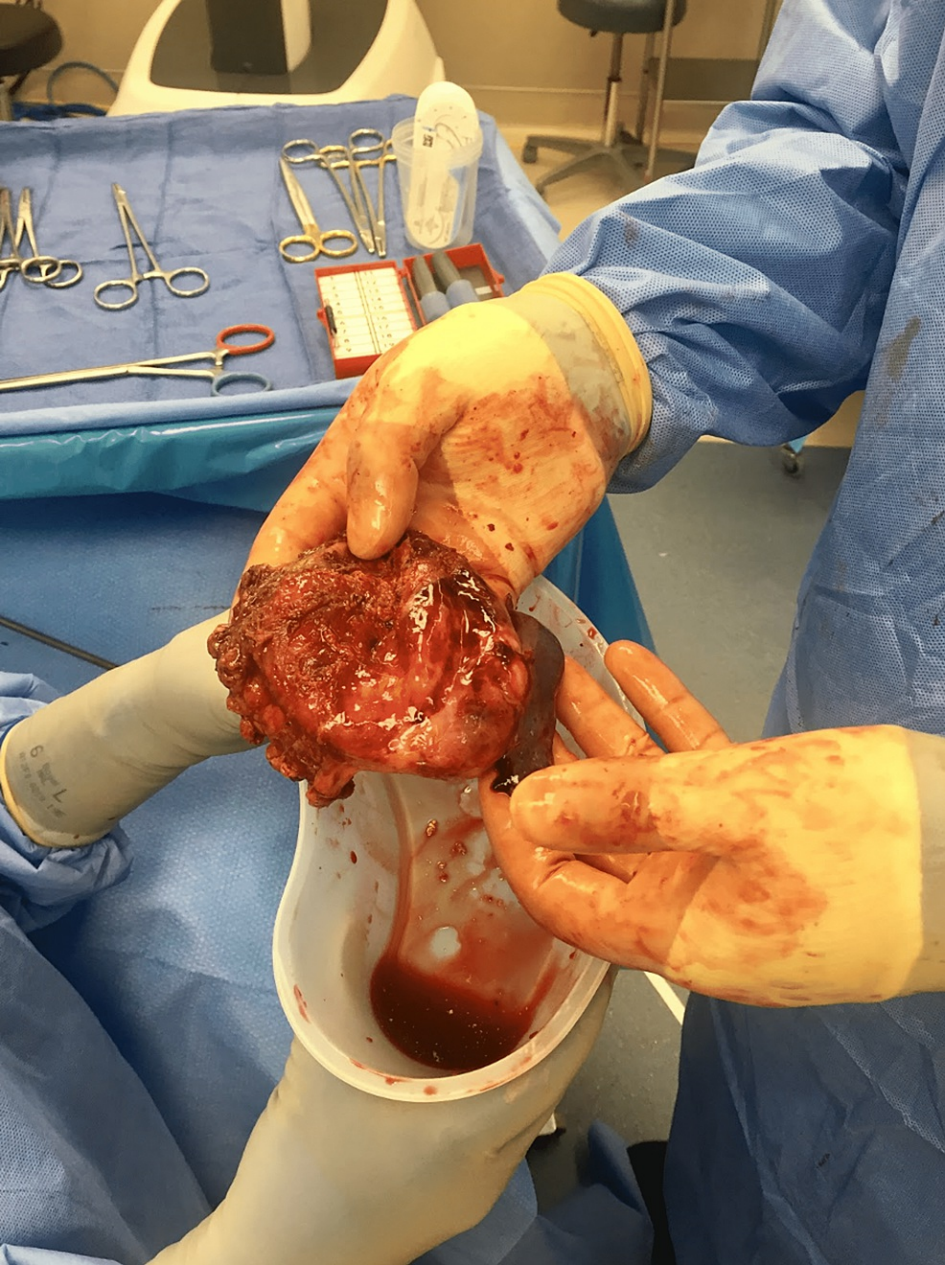
Gross specimen showing mass successfully externalized without spillage

The mass was placed in a bag and the robot was undocked. The bag was externalized and the mass was incised in order to drain the necrotic appearing caseating fluid which allowed for decompression of the mass so it was able to fit through a port site at removal. The mass was successfully externalized without spillage of the fluid contents into the chest (Figure 3). 

**Figure 4:**
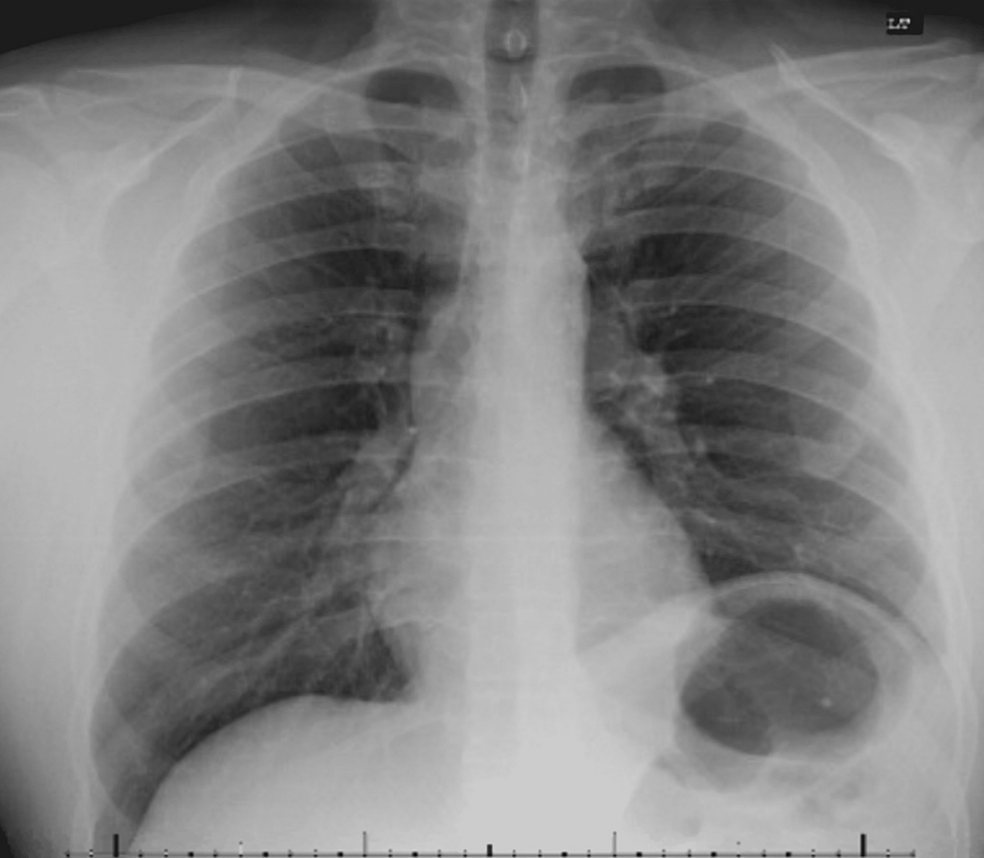
Elevated left hemidiaphragm following surgery

Postoperatively, the patient reported bilateral shoulder pain that was well controlled with pain medication following the operation and he was observed for three days in the hospital. The patient remained vitally stable and did not exhibit any signs or symptoms of an infectious process, and so the antibiotics were deferred at that time. The anterior mediastinal Blake drain was removed on the third day and the patient was safely discharged home in stable condition. Biopsy results of the mass were confirmed with a final pathology diagnosis of mediastinal mature teratoma. The inner aspects of the tissue contained hair. Intraoperative cultures did not detect *P. mirabilis*, likely due to the short antibiotic course and complete drainage during the biopsy. Due to the close adherence of the mass to the left phrenic nerve there was some stretch injury to the nerve. On post-operative day 2, the patient had an elevated left hemidiaphragm that was again detected on repeat CXR one month later (Figure 4). He did not experience any exercise limitations or shortness of breath following the surgery after following up one month later and he only complained of some mild numbness at the incision sites.

## Discussion

Although beneficial, VATS does have many drawbacks including limited maneuverability of thoracoscopic procedures and two-dimensional visualization of the operating field [5]. RATS technology has since evolved from VATS and provides an opportunity to perform highly intricate work with three-dimensional perception using minimally invasive techniques. A retrospective review titled “Robotic video-assisted thoracoscopy: minimally invasive approach for management of mediastinal tumors” illustrated the outcomes of 48 patients undergoing RATS conducted by the same surgeon between the years 2009 and 2013 [5]. The aims of the study included identifying early and late postoperative complications, conversion to the open technique, and tumor recurrence rate. Of the 48 procedures conducted during this timeframe, only one procedure was converted to an open procedure due to the invasiveness of the tumor in that individual patient. Although further studies are needed to compare RATS outcomes with VATS procedures, it is well known that VATS provides much better outcomes and reduced healing time compared to other options, such as an open sternotomy procedure.

## Conclusions

This patient’s presentation involved the intimate association of the teratoma with the phrenic nerve as well as preoperative detection of *P. mirabilis* colonization. The detection of *P. mirabilis* prior to the procedure was also unusual in that the patient did not present with systemic signs of illness. Due to the patient’s hemodynamic stability, there was no indication for postoperative systemic antibiotics. The infection likely resolved following a short course of preoperative antibiotics and complete drainage of the cyst during the biopsy. This was confirmed by the intraoperative cultures. This case has demonstrated the use of the Da Vinci robot as an effective minimally invasive technique in removing masses from the anterior mediastinum. The Da Vinci system allowed for the safe removal of a large mature teratoma that was intimately involved with the phrenic nerve. Due to the nature of teratomas and their variable presentations, this surgical approach must be individually customized to the tumor and the local anatomy. The use of the robotic system should be taken into consideration for all eligible patients with anterior mediastinal teratomas.
